# Association between serologically confirmed COVID-19 infection and cognitive functioning in community dwelling older adults

**DOI:** 10.3389/fneur.2023.1093852

**Published:** 2023-03-21

**Authors:** Sabatini Serena, Pacifico Deborah, Frei Anja, Graf Gwendolyn, Milo A. Puhan, Emiliano Albanese

**Affiliations:** ^1^School of Medicine, University of Nottingham, Nottingham, United Kingdom; ^2^Faculty of Biomedical Sciences, Institute of Public Health (IPH), Università della Svizzera italiana, Lugano, Switzerland; ^3^Epidemiology Biostatistics and Prevention Institute, University of Zurich, Zurich, Switzerland

**Keywords:** cognitive covid, SARS-CoV-2 antibodies, cognitive decline, serological assessment, long covid

## Abstract

**Introduction:**

COVID-19 infection can impact the central nervous system, and is often associated with cognitive decline. However, there are no studies linking serologically confirmed COVID-19 infection with objectively assessed cognitive functioning. We explored whether presence of SARS-CoV-2 antibodies account for variability in participants’ scores on a neuropsychological assessment.

**Methods:**

In this cross-sectional study participants were 657 (mean age = 72.97; SD = 6.07 years; women = 47.7%) individuals randomly selected from the general population of the canton of Zurich and included in the Corona Immunitas study. We conducted serological tests between October 2020 and May 2021 to detect and quantify SARS-CoV-2 antibodies in peripheral venous blood samples. We assessed cognitive function, vaccination status (vaccinated; not vaccinated), number of health conditions, and demographic variables between January and August 2021. We studied the association between seropositivity and global cognitive function and five cognitive domains (language expression, language comprehension, temporal orientation, spatial orientation, and memory) with linear regression models. Based on SARS-CoV-2 antibodies and vaccination status, we stratified participants into three groups: No SARS-CoV-2 antibodies (*N* = 402); SARS-CoV-2 antibodies due to vaccination (*N* = 218); history of SARS-CoV-2 infection and no vaccination (*N* = 37).

**Results:**

In the regression model adjusted for age, sex, educational level, and number of health conditions, compared to those without SARS-CoV-2 antibodies, those with SARS-CoV-2 antibodies due to vaccination had better global cognitive functioning (Standardized beta = 0.10; 95% CI = 0.02; 0.17), and those with SARS-CoV-2 antibodies due to infection had poorer cognitive functioning (Standardized beta = −0.10; 95% CI = −0.18; −0.03). Regarding cognitive domains, compared to those without SARS-CoV-2 antibodies, those with SARS-CoV-2 antibodies due to infection scored more poorly on language comprehension and temporal orientation, and those with SARS-CoV-2 antibodies due to vaccination scored better on memory.

**Discussion:**

By linking serologically confirmed presence of SARS-CoV-2 antibodies to poorer global cognitive functioning in community dwelling older adults we strengthen existing evidence in support of cognitive decline related to COVID-19. Given the large number of infected older adults, and the endurance of the pandemic, our results highlight the need to address COVID-19 related cognitive decline in the clinical and public health areas of prevention, diagnosis, and treatment.

## 1. Introduction

Coronavirus disease (COVID-19) is an infectious disease caused by the SARS-CoV-2 virus. Most common COVID-19 symptoms comprise fever, cough, tiredness, loss of taste or smell whereas less common symptoms comprise sore throat, headache, aches and pains, diarrhea, skin rash, discoloration of fingers or toes, and red or irritated eyes. Few people develop more serious COVID-19 symptoms which comprise difficulty breathing or shortness of breath, loss of speech or mobility, confusion, and chest pain (1). According to the World Health Organization, by end of December 2022, there have been 664,618,938 confirmed cases of COVID-19 worldwide, including 6,722,949 deaths. By end of December 2022 a total of 13,073,712,554 vaccine doses have been administered (2).

Estimations suggest that 20% of individuals who contracted the infection Covid-19 develop post-acute sequelae of COVID-19 (PASC), often referred to as long covid-19 (3–5). PASC refers to the perdurance of COVID-19 symptoms for 12 weeks or longer (6). Milder sequelae of COVID-19 can however continue after having recovered from the viral infection and last up to 18 months (3, 7, 8). Common symptoms of PASC that can persist after 12 weeks are fatigue and headache (9). Another frequent component of PASC, and common long-term consequence of COVID-19 is cognitive impairment, referred to as Cognitive Covid (10). The occurrence of Cognitive Covid is uncertain. Identified cases of Cognitive Covid vary greatly among existing research, ranging between 15% and 80% in COVID-19 patients (11). Nonetheless, numerous studies documented self-reported cognitive difficulties following also laboratory-confirmed COVID-19 infection irrespective of clinical symptoms, particularly in middle-aged and older individuals (12, 13). Subjective cognitive difficulties in COVID-19 patients appear to accurately reflect poorer performance on cognitive tasks (14, 15). Existing studies have also documented how those who contracted COVID-19 scored more poorly on objective cognitive assessments compared to those who did not contract the virus (16). Given that prevention of cognitive decline and Dementia is a global priority (17) and that estimations suggest that 55 million people worldwide are living with dementia (18), further understanding of the link between COVID-19 infection and cognitive functioning is essential.

The COVID-19 infection may cause damage to brain structure and function leading to multi-domain cognitive impairment including memory, executive functions, verbal fluency, processing speed, and visuospatial processing (11, 14, 19, 20). One possible reason for the impact of the COVID-19 infection on cognition could be neurotropism (21–29). SARS-CoV-2 can enter the central nervous system and infect neuronal cells overcoming the blood–brain barrier (21, 30). Moreover, studies using brain magnetic resonance imaging revealed brain microbleeds (30) and cortical hypertension (24) in individuals who contracted COVID-19. Neural damage caused by Covid-19 infection is further confirmed in *post mortem*, neuropathological studies (28). However, it is possible that neurotropism does not occur in all individuals infected with COVID-19, and that the host immune response, including anti-SARS-CoV-2 antibodies may play a modulating role (29). Moreover, substantial evidence in support of neurotropism has not yet been collected and there are few studies even arguing against neurotropism (31–33). Alternative possible explanations for the impact of Covid-19 on cognitive functioning are systemic cerebrovascular changes due to viral-induced inflammation (34).

Flogosis and/or the acquired immune response to infection might underpin cognitive covid. Therefore, irrespective of COVID-19 symptoms, hospitalization, and course of the disease it is important to investigate the association of serologically confirmed COVID-19 infections with cognitive functioning in non-clinical samples. However, while evidence on Cognitive Covid is rapidly expanding in COVID-19 symptomatic patients, epidemiological evidence on the impact on cognitive functioning in individuals with infection-induced SARS-CoV-2 antibodies irrespective of symptoms and severity is lacking. Moreover, observational evidence is very sparse on the impact of the Covid-19 infection on cognitive functioning at older ages, when cognitive decline and dementia sharply increase (34, 35), and most studies have been conducted in samples of hospitalized Covid-19 patients or often with severe COVID-19 symptoms (19, 36). Because the majority of people infected have little or no symptoms and are not hospitalized (12), clinical samples may provide a biased and incomplete picture of the impact of the virus on cognitive function. Spectrum bias may be relevant not only for COVID-19, but also for the sequelae of SARS-CoV-2 infection(s), including on the central nervous system, and thus on brain function, particularly cognition. Epidemiological studies can contribute to complete and unmuddle the clinical picture of Cognitive Covid.

We aimed to study the association between serologically confirmed Covid-19 infection and cognitive function in a large sample of community dwelling individuals aged ≥65 years. We hypothesized that, compared to immunonaïve individuals (i.e., without infection-induced SARS-CoV-2 antibodies) those with a serologically confirmed Covid-19 infection have poorer cognitive function. Because more educated individuals are more favorable toward COVID-19 vaccination (37–39), and education can be a proxy of cognitive functioning in older age (40), we also hypothesized that, compared to those who are not vaccinated, those who are vaccinated against COVID-19 have better cognitive functioning. A previous study has indeed found that individuals aged 16–95 with poorer cognitive functioning report higher hesitancy toward uptake of COVID-19 vaccine (41).

## 2. Methods

### 2.1. Recruitment and participants

This study used data from the Corona Immunitas study (42) from the Canton of Zurich, Switzerland. Randomly selected individuals from the general population were invited to take part in the study *via* letter or email. Individuals aged ≥65 years who agreed to participate in Corona Immunitas were asked to take part also in an additional study focusing on cognition (SwissDEM). To this additional study 657 individuals, who self-reported lack of diagnosis of dementia, participated and comprised the current study sample. Participants’ mean age was 72.97 years (SD = 6.07, range: 65–93). Slightly below half of participants were women (47.7%). On average participants reported one clinically diagnosed health conditions. On average, participants were cognitively healthy as indicated by the mean score on the CSI’D′ in the whole sample (*M* = 33.79; SD = 1.35; range = 20.17–35.00). Further details on the demographic characteristics and number of health conditions for the study sample and by immunization and infection status are reported in Table 1.

**Table 1:** Descriptive statistics for sociodemographic variables and health conditions for the overall study sample and across subgroups based on presence of COVID-19 antibodies.

Variables	Overall study sample (*N* = 657)	No SARS-CoV-2 antibodies (*N* = 402)	SARS-CoV-2 antibodies due to vaccination (*N* = 218)	Serologically confirmed history of SARS-CoV-2 infection and no vaccination (*N* = 37)	Value of *p* derived from chi-squared test/ANOVA comparing the three study groups
Age, *M* (SD)	72.97 (6.08)	72.97 (6.00)	72.76 (5.85)	74.22 (7.90)	0.4037
Sex, female n (%)	313 (47.7)	194 (48.3)	102 (46.8)	17 (46.0)	0.707
Education, *n* (%)					
Primary school	12 (1.8)	10 (2.5)	2 (0.9)	0 (0)	<0.001
Secondary school	285 (43.4)	187 (46.5)	79 (36.2)	19 (51.4)	
High school	115 (17.5)	62 (15.4)	47 (21.6)	6 (16.2)	
University certificate	245 (37.3)	143 (35.6)	90 (41.3)	12 (32.4)	
Number of health conditions, *M* (SD)	0.87 (1.12)	0.68 (1.02)	1.25 (1.22)	0.59 (1.12)	<0.001
Zero health conditions, *n* (%)	334 (50.8)	239 (59.5)	70 (32.1)	25 (67.6)	
One health condition, *n* (%)	170 (25.9)	87 (21.6)	76 (34.9)	7 (18.9)	
Two health conditions, *n* (%)	89 (13.6)	53 (13.2)	34 (15.6)	2 (5.4)	
Three health conditions, *n* (%)	43 (6.5)	14 (3.5)	27 (12.4)	2 (5.4)	
Four health conditions, *n* (%)	14 (2.1)	5 (1.2)	9 (4.1)	0 (0)	
Five health conditions, *n* (%)	6 (0.9)	4 (1.0)	1 (0.5)	1 (2.7)	
Six health conditions, *n* (%)	1 (0.2)	0 (0)	1 (0.5)	0 (0)	

ANOVA, analysis of variance.

### 2.2. Sociodemographic and cognitive data collection

Data collection for sociodemographic variables, health conditions, and cognitive assessments took place in person usually at the University of Zurich or, if required, also at the participants’ home between January and August 2021. More specifically, 49 participants were assessed in January 2021, 98 in February 2021, 87 in March 2021, 75 in April 2021, 76 in May 2021, 132 in June 2021, 106 in July 2021, and 34 in August 2021. Standard training preceded all interviews, following standard 1,066 procedures (43). RedCap (i.e., Research Electronic Data Capture) (44, 45) on dedicated tablets with data encryption was used for data collection.

### 2.3. Blood processing

To measure seropositivity to SARS-CoV-2 peripheral venous blood samples were collected between October 2020 and May 2021. For 47% of participants data was collected in 2020 and for the remaining 53% data was collected in 2021.

All participants provided written informed consent before blood-sampling for the Corona Immunitas Zurich study and additional consent for participation in the SwissDEM study. Ethical approval was obtained from the local Ethics committee Zurich (Swiss BASEC Registration No 2020–01247).

As data was collected at the beginning of the vaccination campaign in Zurich, this made it possible to obtain a sample comprising individuals non-infected with SARS-CoV-2, individuals infected with SARS-CoV-2, and individuals vaccinated against SARS-CoV-2. Hence, combining information from serological testing (presence versus absence of anti-N SARS-CoV-2 antibodies) with self-reported vaccination status (vaccinated versus not vaccinated) we generated a variable called immunization status and comprising three categories: 0 = No SARS-CoV-2 antibodies (neither vaccination nor infection); 1 = SARS-CoV-2 antibodies due to vaccination (only anti-S and vaccinated); 3 = SARS-CoV-2 antibodies due to infection (anti-N, and not vaccinated). Among study participants 61.2% had no SARS-CoV-2 antibodies, 33.2% a relevant proportion had SARS-CoV-2 antibodies due to vaccination, and only 5.6% had SARS-CoV-2 antibodies due to infection.

### 2.4. Variables and measures

#### 2.4.1. Cognitive functioning

We assessed cognitive function and impairment with the Community Screening Instrument for Dementia (CSI’D′), participant part (46). The CSI’D′ is a widely used, culturally unbiased cognitive battery. The total score is an indicator of global cognitive functioning and is calculated by summing its 35 items. Lower total scores (possible range: 0–35) indicate worse general cognitive functioning or cognitive impairment. A clinical cut off score is not available and generally not used in empirical studies. We also calculated the CSI’D′ subscales scores of language expression (possible range: 0–4), language comprehension (possible range: 0–2), temporal orientation (possible range: 0–4), spatial orientation (possible range: 0–4), and memory (possible range: 0–13) by computing their respective items. Language expression, temporal orientation, spatial orientation, and memory all comprise four items each whereas language comprehension comprises two items.

#### 2.4.2. Demographic and health characteristics

Demographic variables comprised age (in years), sex (women = 1; men = 2), and educational level. Educational level comprised four categories: primary school; secondary school; high school; and university certificate. Participants reported previous clinical diagnosis of cancer, diabetes, immunological disorders, hypertension, cardiovascular disease, respiratory diseases, allergy, and other diseases. A count variable was created for number of health conditions.

#### 2.4.3. Serological testing

To assess seropositivity to SARS-CoV-2, we used a previously validated Luminex assay for anti-SARS-CoV-2 total immunoglobulins, purposely developed for population-based serosurveys [for further details see (47, 48)]. We measured antibodies targeting the spike and nucleocapside proteins of the virus (i.e., anti-N SARS-CoV-2 antibodies). Whereas nucleocapside proteins are developed only following natural infection, spike antibodies can be developed following both natural infection and vaccination, facilitating distinction between infection-and/or vaccine-induced immunity.

### 2.5. Statistical analysis

We reported descriptive statistics (Mean, M and Standard Deviation, SD for numeric variables and number, n and percentage, % for categorical and ordinal variables) of study variables including sociodemographic characteristics for the overall study sample and for those with no SARS-CoV-2 antibodies; SARS-CoV-2 antibodies due to vaccination; and SARS-CoV-2 antibodies due to infection.

To explore whether levels of cognitive functioning differ among individuals with different immunization status we conducted unadjusted and adjusted (for age, sex, educational level, and number of health conditions) linear regression models with immunization status (no SARS-CoV-2 antibodies; SARS-CoV-2 antibodies due to vaccination; SARS-CoV-2 antibodies due to infection) as the predictive variable and participants’ total and subscales scores (language expression, language comprehension, temporal orientation, spatial orientation, and memory) on the CSI’D′ as outcome. We conducted complete case analysis. We reported unstandardized and standardized regression coefficients (*ß*, effects sizes) to quantify the associations. Standardized coefficients ≤0.09 indicated negligible effects, between 0.10 and 0.29 indicated small effects, between 0.30 and 0.49 indicated moderate effects, and ≥0.50 were indicated large effects (49). We conducted analyses in STATA version 16.1 (50).

## 3. Results

### 3.1. Descriptive statistics for cognitive functioning

Mean score on the CSI’D′ was 33.73 among those with no SARS-CoV-2 antibodies; 34.03 among those with SARS-CoV-2 antibodies due to vaccination; and 33.06 in those with SARS-CoV-2 antibodies due to infection. Mean score on language expression was 3.99 among those with no SARS-CoV-2 antibodies; 4 among those with SARS-CoV-2 antibodies due to vaccination; and 4 in those with SARS-CoV-2 antibodies due to infection. Mean score on language comprehension was 1.98 among those with no SARS-CoV-2 antibodies; 1.98 among those with SARS-CoV-2 antibodies due to vaccination; and 1.92 in those with SARS-CoV-2 antibodies due to infection. Mean score on temporal orientation was 3.97 among those with no SARS-CoV-2 antibodies; 3.99 among those with SARS-CoV-2 antibodies due to vaccination; and 3.84 in those with SARS-CoV-2 antibodies due to infection. Mean score on spatial orientation was 3.94 among those with no SARS-CoV-2 antibodies; 3.95 among those with SARS-CoV-2 antibodies due to vaccination; and 3.97 in those with SARS-CoV-2 antibodies due to infection. Mean score on memory was 11.68 among those with no SARS-CoV-2 antibodies; 12.22 among those with SARS-CoV-2 antibodies due to vaccination; and 11.14 in those with SARS-CoV-2 antibodies due to infection. Further details on cognitive functioning in the overall sample and on differences in the cognitive functioning of study groups are reported in Table 2.

**Table 2:** Descriptive statistics for cognitive functioning for the overall study sample and across subgroups based on presence of COVID-19 antibodies.

Variables	Overall study sample (*N* = 657)	No SARS-CoV-2 antibodies (*N* = 402)	SARS-CoV-2 antibodies due to vaccination (*N* = 218)	Serologically confirmed history of SARS-CoV-2 infection and no vaccination (*N* = 37)	Value of *p* derived from ANOVA comparing the three study groups
Global cognitive functioning, *M* (SD; range)	33.79 (1.35; 20–35)	33.73 (1.28; 24–35)	34.03 (1.13; 28–35)	33.07 (2.51; 20–35)	<0.001
Language expression, *M* (SD; range)	4.0 (0.07; 3–4)	3.99 (0.09; 3–4)	4 (0; 4–4)	4 (0; 4–4)	0.3856
Language comprehension, *M* (SD; range)	1.98 (0.17; 0–2)	1.98 (0.15; 0–2)	1.98 (0.15; 1–2)	1.92 (0.36; 0–2)	0.0893
Temporal orientation, *M* (SD; range)	3.97 (0.20; 1–4)	3.97 (0.18; 2–4)	3.99 (0.10; 3–4)	3.84 (0.55; 1–4)	<0.001
Spatial orientation, *M* (SD; range)	3.95 (0.23; 3–4)	3.94 (0.24; 3–4)	3.95 (0.22; 3–4)	3.97 (0.16; 3–4)	0.6658
Memory, *M* (SD; range)	11.83 (1.48; 4–13)	11.68 (1.54; 5–13)	12.22 (1.16; 7–13)	11.14 (2.03; 4–13)	<0.001

ANOVA, analysis of variance.

### 3.2. Associations between immunization status and cognition

Results from unadjusted and adjusted (for age, sex, educational level, and number of health conditions) linear regression models with infection status (no SARS-CoV-2 antibodies; SARS-CoV-2 antibodies due to vaccination; SARS-CoV-2 antibodies due to infection) as the predictor of participants’ scores on the CSI’D′ are reported in Table 3. In the adjusted model, compared to those without SARS-CoV-2 antibodies, those with SARS-CoV-2 antibodies due to vaccination had better global cognitive functioning (*ß*, Standardized beta = 0.10; 95% CI = 0.02; 0.17), and those with SARS-CoV-2 antibodies due to infection had poorer cognitive functioning (Standardized beta = −0.10; 95% CI = −0.18; −0.03). Moreover, in the adjusted model, compared to those without SARS-CoV-2 antibodies, those with SARS-CoV-2 antibodies due to infection scored more poorly on language comprehension (Standardized beta = −0.08; 95% CI = −0.16; −0.004) and temporal orientation (Standardized beta = −0.15; 95% CI = −0.22; −0.07), and those with SARS-CoV-2 antibodies due to vaccination scored better on memory (Standardized beta = 0.15; 95% CI = 0.08; 0.23). We found no statistical significance for serologically-confirmed infection or vaccination status and the remaining cognitive domains scores of language expression and spatial orientation (see Table 4).

**Table 3:** Infection status as predictor of general cognitive functioning.

	General cognitive functioning as outcome			
	Unadjusted		Adjusted	
	Unstandardized beta (95% CI)	Standardized beta (95% CI)	Unstandardized beta (95% CI)	Standardized beta (95% CI)
Immunization status				
Reference group (no SARS-CoV-2 antibodies)				
SARS-CoV-2 antibodies due to vaccination	**0.30 (0.08; 0.52)**	**0.10 (0.03; 0.18)**	**0.28 (0.06; 0.50)**	**0.10 (0.02; 0.17)**
Serologically confirmed history of SARS-CoV-2 infection and no vaccination	**−0.67 (−1.12; −0.22)**	**−0.11 (−0.19; −0.04)**	**−0.60 (−1.03; −0.16)**	**−0.10 (−0.18; −0.03)**
Age			**−0.05 (−0.07; −0.04)**	**−0.24 (−0.31; −0.16)**
Sex			−0.12 (−0.33; 0.08)	−0.05 (−0.12; 0.03)
Educational level			0.05 (−0.06; 0.16)	0.03 (−0.04; 0.11)
Number of health conditions			0.002 (−0.09; 0.09)	0.001 (−0.07; 0.08)

Adjusted for age, sex, educational level, and number of health conditions. Statistically significant values are printed in bold.

**Table 4:** Infection status as predictor of cognitive domains.

	Language expression as outcome			
	Unadjusted		Adjusted	
	Unstandardized beta (95% CI)	Standardized beta (95% CI)	Unstandardized beta (95% CI)	Standardized beta (95% CI)
Immunization status				
Reference group (no SARS-CoV-2 antibodies)				
SARS-CoV-2 antibodies due to vaccination	0.01 (−0.004; 0.02)	0.05 (−0.03; 0.13)	0.01 (−0.003; 0.02)	0.06 (−0.02; 0.14)
Serologically confirmed history of SARS-CoV-2 infection and no vaccination	0.01 (−0.02; 0.03)	0.03 (−0.05; 0.10)	0.01 (−0.01; 0.03)	0.03 (−0.05; 0.11)
Age			**−0.001 (−0.002; −0.001)**	**−0.12 (−0.20; −0.04)**
Sex			−0.001 (−0.01; 0.01)	−0.01 (−0.09; 0.07)
Educational level			−0.002 (−0.01; 0.004)	−0.02 (−0.10; 0.05)
Number of health conditions			−0.002 (−0.01; 0.003)	−0.03 (−0.11; 0.04)
	**Language comprehension as outcome**			
	Unadjusted		Adjusted	
	Unstandardized beta (95% CI)	Standardized beta (95% CI)	Unstandardized beta (95% CI)	Standardized beta (95% CI)
Immunization status				
Reference group (no SARS-CoV-2 antibodies)				
SARS-CoV-2 antibodies due to vaccination	−0.01 (−0.03; 0.02)	−0.02 (−0.09; 0.06)	−0.01 (−0.04; 0.02)	−0.02 (−0.10; 0.06)
Serologically confirmed history of SARS-CoV-2 infection and no vaccination	**−0.06 (−0.12; −0.01)**	**−0.09 (−0.16; −0.01)**	**−0.06 (−0.11; −0.002)**	**−0.08 (−0.16; −0.004)**
Age			**−0.01 (−0.01; −0.003)**	**−0.17 (−0.24; −0.10)**
Sex			−0.01 (−0.04; 0.01)	−0.04 (−0.12; 0.03)
Educational level			−0.001 (−0.01; 0.01)	−0.003 (−0.08; 0.07)
Number of health conditions			0.01 (−0.001; 0.02)	0.07 (−0.01; 0.15)
	Temporal orientation as outcome			
	Unadjusted		Adjusted	
	Unstandardized beta (95% CI)	Standardized beta (95% CI)	Unstandardized beta (95% CI)	Standardized beta (95% CI)
Immunization status				
Reference group (no SARS-CoV-2 antibodies)				
SARS-CoV-2 antibodies due to vaccination	0.02 (−0.01; 0.05)	0.05 (−0.03; 0.12)	0.02 (−0.01; 0.06)	0.05 (−0.03; 0.13)
Serologically confirmed history of SARS-CoV-2 infection and no vaccination	**−0.13 (−0.20; −0.06)**	**−0.15 (−0.22; −0.07)**	**−0.13 (−0.19; −0.06)**	**−0.15 (−0.22; −0.07)**
Age			**−0.01 (−0.01; −0.002)**	**−0.14 (−0.21; −0.06)**
Sex			−0.02 (−0.05; 0.01)	−0.04 (−0.12; 0.03)
Educational level			−0.01 (−0.03; 0.01)	−0.04 (−0.12; 0.04)
Number of health conditions			0.001 (−0.01; 0.01)	0.004 (−0.07; 0.08)
	Spatial orientation as outcome			
	Unadjusted		Adjusted	
	Unstandardized beta (95% CI)	Standardized beta (95% CI)	Unstandardized beta (95% CI)	Standardized beta (95% CI)
Immunization status				
Reference group (no SARS-CoV-2 antibodies)				
SARS-CoV-2 antibodies due to vaccination	0.01 (−0.03; 0.05)	0.02 (−0.06; 0.10)	0.02 (−0.02; 0.05)	0.03 (−0.05; 0.11)
Serologically confirmed history of SARS-CoV-2 infection and no vaccination	0.03 (−0.04; 0.11)	0.03 (−0.04; 0.11)	0.04 (−0.04; 0.11)	0.04 (−0.04; 0.11)
Age			−0.003 (−0.01; 0.0002)	−0.07 (−0.15; 0.01)
Sex			−0.03 (−0.06; 0.01)	−0.06 (−0.14; 0.02)
Educational level			−0.01 (−0.03; 0.01)	−0.05 (−0.13; 0.03)
Number of health conditions			−0.01 (−0.02; 0.01)	−0.04 (−0.12; 0.04)
	Memory as outcome			
	Unadjusted		Adjusted	
	Unstandardized beta (95% CI)	Standardized beta (95% CI)	Unstandardized beta (95% CI)	Standardized beta (95% CI)
Immunization status				
Reference group (no SARS-CoV-2 antibodies)				
SARS-CoV-2 antibodies due to vaccination	**0.54 (0.30; 0.78)**	**0.17 (0.10; 0.24)**	**0.48 (0.24; 0.72)**	**0.15 (0.08; 0.23)**
Serologically confirmed history of SARS-CoV-2 infection and no vaccination	**−0.55 (−1.04; −0.06)**	**−0.09 (−0.16; −0.01)**	−0.46 (−0.93; 0.01)	−0.07 (−0.14; 0.001)
Age			**−0.06 (−0.08; −0.04)**	**−0.25 (−0.32; −0.18)**
Sex			**−0.29 (−0.50; −0.07)**	**−0.10 (−0.17; −0.02)**
Educational level			**0.17 (0.06; 0.29)**	**0.11 (0.04; 0.18)**
Number of health conditions			0.02 (−0.08; 0.11)	0.01 (−0.06; 0.09)

Adjusted for age, sex, educational level, and number of health conditions. Statistically significant values are printed in bold.

## 4. Discussion

We quantified the impact of COVID-19 infection on cognitive function in community dwelling older people using previously validated serological tests and in-person neuropsychological assessments, respectively. Study results showed that compared to those with no SARS-CoV-2 antibodies, those with SARS-CoV-2 antibodies due to infection had slightly poorer general cognitive functioning whereas those with SARS-CoV-2 antibodies due to vaccination had slightly better global cognitive functioning. Moreover, compared to those without SARS-CoV-2 antibodies, those with SARS-CoV-2 antibodies due to infection scored more poorly on language comprehension and temporal orientation, and those with SARS-CoV-2 antibodies due to vaccination scored better on memory. Though statistically significant, the small size of effects suggests that COVID-19 infection may have a relatively small negative impact on the cognitive functioning of community-dwelling older individuals without dementia and especially on their language comprehension and temporal orientation abilities. Our findings are aligned with, and of comparable magnitude of existing evidence linking Covid-19 infection with poorer objectively assessed cognitive functioning in clinical samples (16). Our results extend previous evidence in middle-aged individuals to older age, a critical period for cognitive impairment and decline.

Previous research linking COVID-19 infection with cognitive functioning is limited to clinical samples, and focused on hospitalized COVID-19 patients with severe symptoms. Our study suggests that the association of COVID-19 infection with poorer overall cognitive functioning may exist also in a more heterogeneous sample of community-dwelling middle aged and older adults without dementia. In line with and support of our results, another study also found a link between COVID-19 infection and poorer cognitive functioning among non-hospitalized individuals with COVID-19 (12).

One of the possible explanations for our results is that the virus neurotropism and ensuing brain damage, which in turn may lead to cognitive difficulties, may not be related to systemic COVID-19 symptoms (21, 30). However, there are also studies suggesting this possible explanation is unlikely (31–33), and that drivers of cognitive impairment in individuals recovering from COVID-19 remain largely unknown (51, 52). Alternative possible explanations for the impact of COVID-19 on cognitive functioning are systemic cerebrovascular changes due to viral-induced inflammation (53). Further evidence is warranted but our findings may have considerable public health implications because COVID-19 infections greatly outnumber symptomatic, severe, and hospitalized COVID-19 cases (54). Prevention of SARS-CoV-2 infections may be important to reduce the overall dementia attributable fraction that might be due to COVID-19, which is a function of infection-induced seroprevalence (55).

It is worth noting that different from previous studies that reported scores below the cut-off for normal cognitive functioning among those infected with COVID-19, our sample comprises older adults without self-reported diagnosis of dementia and who scored high on the CSI’D′. In our study sample the group with SARS-CoV-2 antibodies scored within normal ranges of overall cognitive scores (8), but these scores were statistically significantly lower than those of the group of participants with no SARS-CoV-2 antibodies. The mean difference in the CSI’D′ (46), which total score can range between 0 and 35, was −0.60. So, while the impact of COVID-19 infection over the cognitive functioning of older adults without dementia may be lower than that of other risks factors (e.g., smoking, hypertension) for cognitive decline (34, 56), the size of the impact on cognitive performance equated to the size of the effect of Aducanumab (i.e., the only approved drug against Alzheimer’s disease) on cognitive functioning in the ADAS-cog 70 point scale (57).

Because Cognitive Covid is a new construct, and evidence is sparse not only on its occurrence but also on its causal mechanisms, prognosis and impact in the mid-and long-term are unknown. Cognitive Covid may represent an “acute” symptom caused by the virus. Nevertheless, there is some evidence documenting how cognitive difficulties in some of the first cases of COVID-19 endured up to 1 year, and with null viral load (12). In fact, SARS-CoV-2 may cause an irreversible decline in cognitive functioning which may or not further progress after the complete clearance of the virus. Cognitive Covid could be the beginning of a trajectory of cognitive decline potentially due to the chronicity of the inflammation caused by the virus (51, 58).

We combined information on serological assessment with self-reported vaccination status to discern between those with SARS-CoV-2 antibodies due to infection and those with SARS-CoV-2 antibodies due to vaccination. We found that older adults who vaccinated against Covid-19 had slightly better cognitive functioning, and in particular memory, compared to those who did not vaccinate against COVID-19 (and were not infected). Contrary to our expectations and to existing evidence suggesting that less-well educated individuals are less likely to get vaccinated (37–39), educational level did not confound the association between vaccination status and overall cognitive function. However, vaccinated individuals were statistically significantly better educated than the two remaining study groups.

This study has several strengths. First, differently from all other studies on the topic that relied on self-reported COVID-19 symptoms or positive SARS-CoV-2 test (PCR test or antigen rapid test) as indicator of COVID-19 infection, we used a serological test to detect anti-SARS-CoV-2 antibodies. Spectrum bias is highly unlikely. Second, we conducted in-person cognitive assessment. Self-reported, subjective cognitive difficulties reported by COVID-19 patients have been previously used but are prone to information biased, and may not relate to objective cognitive function (59). Third, the study sample comprises randomly recruited individuals in old and advanced old age, who were underrepresented in previous studies on the topic. Fourth, different from many previous studies that selectively focused on hospitalized individuals with COVID-19, we focused on a representative sample of community dwelling older adults without self-reported dementia. Fifth, in the analyses we controlled for the potential confounding effects of health conditions and age on cognition. Sixth, data was collected early in the vaccination campaign, allowing the study of a mixed population of vaccinated and non-vaccinated individuals.

This study has also several limitations too. Even though our measure of cognitive functioning (46) is more sensitive than those used in most previous studies (19, 60), such as the Mini Mental State Examination (61) and the Montreal Cognitive Assessment (62), it is not free from bias. The CSI’D′ participant part (46) was designed as a screening tool for pathological cognitive decline. A ceiling effect may not be excluded because our participants had an overall high cognitive functioning. Our results may be an underestimate of the true effect of COVID-19 infection on cognitive functioning. Next, issues of directionality due to the cross-sectional design seem implausible but cannot be excluded also because of the lack of pre-pandemic cognitive assessments that did not allow to capture changes in cognitive functioning prior and after infection. Moreover, although we had information about self-reported diagnosis of dementia and all participants scored high on the CSI’D′ – indicating cognitive health - we cannot completely exclude that a minority of participants may have had cognitive decline.

Although only 5.6% of the study sample had SARS-CoV-2 antibodies due to infection we could detect a statistically significant effect on cognition. That the temporal span between the cognitive assessment and the blood test varied among participants is a potential additional limitation. However, considering that in person assessment of cognitive functioning is more time consuming than self-reporting used in previous studies, our study retained a good balance between internal validity and feasibility. A final limitation of our study is that we did not consider SARS-CoV-2 variants, which may have differential pathogenicity, neurotropism, and immune escape potential (63–65). However, we collected blood samples before the Omicron variants appeared, and mainly during the concomitant presence of the alpha and delta variants, which share similar infectiousness and pathogenic characteristics (64).

## 5. Conclusion

We found preliminary and novel epidemiological evidence in support of Cognitive Covid, by showing that, in a sample of community dwelling individuals aged 65 years and over and without dementia, serologically-confirmed COVID-19 infection was cross-sectionally associated with lower cognitive performance, especially in the domains of language comprehension and temporal orientation. We extend evidence from individuals infected with COVID-19 to the much larger population of those who got infected with the virus and were pauci-or a-symptomatic. The scale of Cognitive Covid may be larger than previously thought, and may even slightly contribute to worsen the expected increases in dementia occurrence, and require recalculating projections based on demographic changes only. Given the preliminary nature of our results, further cognitive assessment of older people with evidence of COVID-19 infection is warranted to monitor trajectory of cognitive decline, and accordingly implement secondary prevention interventions at large scale.

## Data availability statement

The original contributions presented in the study are included in the article/supplementary material, further inquiries can be directed to the corresponding author.

## Ethics statement

The studies involving human participants were reviewed and approved by Swiss BASEC Registration No 2020–01247 University of Italian Switzerland and University of Zurich. The patients/participants provided their written informed consent to participate in this study.

## Author contributions

SS performed the data analyses and drafted the manuscript. EA contributed to the data collection and co-edited the manuscript. DP, AF, and MP contributed to the data collection and provided comments to the manuscript. DP and GG contributed in terms of data collection. All authors contributed to the article and approved the submitted version.

## Funding

This study is part of the Corona Immunitas research network, coordinated by the Swiss School of Public Health (SSPH+), and funded by fundraising of SSPH+ that includes funds of the Swiss Federal Office of Public Health and private funders (ethical guidelines for funding stated by SSPH+ were respected), by funds of the canton of Zurich, and by institutional funds of the university. SwissDEM was funded by the Swiss National Science Foundation (SNSF), 320030_184794. The funders had no role in the design and conduct of the study; collection, management, analysis, and interpretation of the data; preparation, review, or approval of the manuscript; and decision to submit the manuscript for publication.

## Conflict of interest

The authors declare that the research was conducted in the absence of any commercial or financial relationships that could be construed as a potential conflict of interest.

## Publisher’s note

All claims expressed in this article are solely those of the authors and do not necessarily represent those of their affiliated organizations, or those of the publisher, the editors and the reviewers. Any product that may be evaluated in this article, or claim that may be made by its manufacturer, is not guaranteed or endorsed by the publisher.
